# Fast Mapping of Global Protein Folding States by Multivariate NMR: A GPS for Proteins

**DOI:** 10.1371/journal.pone.0010262

**Published:** 2010-04-21

**Authors:** Anders Malmendal, Jarl Underhaug, Daniel E. Otzen, Niels C. Nielsen

**Affiliations:** 1 Center for Insoluble Protein Structures, Interdisciplinary Nanoscience Center and Department of Chemistry, Aarhus University, Aarhus, Denmark; 2 Center for Insoluble Protein Structures, Interdisciplinary Nanoscience Center and Department of Molecular Biology, Aarhus University, Aarhus, Denmark; Griffith University, Australia

## Abstract

To obtain insight into the functions of proteins and their specific roles, it is important to establish efficient procedures for exploring the states that encapsulate their conformational space. Global Protein folding State mapping by multivariate NMR (GPS NMR) is a powerful high-throughput method that provides such an overview. GPS NMR exploits the unique ability of NMR to simultaneously record signals from individual hydrogen atoms in complex macromolecular systems and of multivariate analysis to describe spectral variations from these by a few variables for establishment of, and positioning in, protein-folding state maps. The method is fast, sensitive, and robust, and it works without isotope-labelling. The unique capabilities of GPS NMR to identify different folding states and to compare different unfolding processes are demonstrated by mapping of the equilibrium folding space of bovine α-lactalbumin in the presence of the anionic surfactant sodium dodecyl sulfate, SDS, and compare these with other surfactants, acid, denaturants and heat.

## Introduction

Life depends on proteins interacting mutually and with other molecules in complex patterns in time and space. This web of interactions may be described in terms of interactomes of the proteins [1], [2], [3], but biological function is intimately linked to the structural/dynamic response of the proteins to these interactions. In this perspective, it is important to establish procedures to explore the states that encapsulate the conformational space of the proteins.

Here we introduce Global Protein folding State mapping by multivariate NMR (GPS^1^ NMR) for fast establishment of, and navigation in, protein folding-state maps when proteins are exposed to perturbations, such as changing thermodynamic or solvent conditions, or molecular interactions. The folding maps provide a structural overview which complements interactomic networks, direct structural biologists to states that reflect the conformational space of proteins, and identify optimal conditions for studies of protein complexes and assemblies such as membrane proteins or fibrils.

GPS NMR is based on ^1^H liquid-state NMR in combination with multivariate analysis. Simple 1D ^1^H spectra simultaneously provide chemical shifts of ^1^H located all around an organic molecule, the values of which are sensitive to changes in the local environment. Thus, virtually any perturbation of the protein can be detected, and through appropriate data analysis a wealth of information can be extracted and used to map protein interactions or folding states. This is important when comparing with other biophysical techniques, such as circular dichroism or fluorescence spectroscopy, where only the global fold or a few optically active probes are used to examine folding and interactions that may concern other regions than those probed. Multivariate analysis allows the changes in the local environment of all ^1^H within a molecule to be summarized in a way that provides an easily accessible overview and defines the different states that are sampled. In contrast to classical analysis of NMR data which involves time-consuming assignment prior to data evaluation, unsupervised multivariate analyses such as principal component analysis (PCA) [4] is extremely fast and can be applied to the data without manual pre-treatment. Furthermore, it allows analysis of data at a resolution or sensitivity where classical analysis is useless. This allows for high-throughput (seconds to minutes per experiment) and small amounts of sample (mM concentration). The method also works well for complex mixtures and multiple processes, and does not rely on isotope labeling. GPS NMR thus differs markedly from previous combinations of protein NMR with multivariate analysis involving isotope-labeling and/or advanced spectral assignment protocols [5], [6].

Multivariate analysis facilitates separation of the different contributions to the variation between spectra by reducing the dimensionality of a data set while retaining the primary information. The principal components (PCs) are uncorrelated, and ordered by the amount of information they contain. Each PC is described by a “loading” vector, *i.e.* positive/negative peaks as a function of chemical shift, and “scores” that describe the relative contribution of the loading vector to each spectrum. The processes that occur *e.g.* as a ligand is added are well described by a few PCs. Since the number of processes is much lower than the number of frequencies measured, this applies also in the case of severely overlapping resonances. There is thus no need for unique protein or ligand signals to follow the changes in the different populations.

It is important to note that even if the spectral changes are described by only a few PCs, the loadings describe the different spectral changes that occur and the scores describe the different samples in terms of these changes. Ideally, the only information that is lost in this procedure is random spectral variations, i.e. noise. However, if a detailed analysis of the macromolecular structure and dynamics is needed, the full potential of macromolecular NMR methods should be focused on the states identified using GPS NMR.

Here we demonstrate GPS NMR for folding-state mapping of the structural transitions in bovine α-lactalbumin (BLA). BLA is a mixed α/β Ca^2+^-binding protein containing 4 disulfide bonds, and has been an intensively studied model for protein folding [7], [8]. It has been shown that BLA is remarkably sensitive to surfactants [9]. We follow the unfolding of BLA by surfactants, acid, denaturants and heat, and show the capabilities of GPS NMR to identify different folding states and to compare different unfolding processes.

## Materials and Methods

### Sample preparation

Unless otherwise stated 1.2 mM apo BLA was dissolved in a 20 mM sodium phosphate buffer at pH 7.0 containing 5 mM EDTA, 10% D_2_O for frequency lock and 0.25 mM sodium 2,2-dimethyl-2-silapentane-5-sulfonate, DSS, for chemical shift reference. The titrations involved 10–20 additions of: SDS (0–42 mM), C_7_PC (0–56 mM), DM (0–39 mM), GdmCl (0–3.3 M), TFE (0–50%) to BLA in the NMR tube. pH titrations were performed on unbuffered solutions by addition of HCl (pH 8.5–2.2) and NaOH (pH 1.4–6.2). Spectra of apo BLA were acquired at increasing temperatures (10–60, 10°C). Generally spectra were acquired at 700.09 MHz. The SDS titration was also performed at 400.13 MHz. An additional SDS titration was performed at 799.30 MHz on a sample containing 12 µM BLA.

### NMR experiments

NMR spectra were acquired at 25°C on a Bruker Avance-II 700 (700.09 MHz) or Avance 400 (400.13 MHz) spectrometers using standard liquid-state probes with z-gradients. For each addition a WATERGATE spectrum [10] was acquired (32768 points, 128 scans, spectral width 17.96 ppm). Prior to acquisition, the sample was allowed to equilibrate for 5 min in the spectrometer, followed by quick shimming. Similarly experiments were recorded as the temperature was increased from 10 to 60°C in steps of 1°C. Additional spectra were acquired with 4 scans on a Bruker Avance 800 spectrometer (799.30 MHz), equipped with a cryogenic probe.

### Data analysis

The spectra were processed and binned using iNMR (www.inmr.net). An exponential line-broadening of 5 Hz was applied prior to Fourier transformation (50 Hz for the 12 µM sample). All spectra were referenced to DSS at 0.0 ppm, automatically phased and baseline corrected. Data reduction was accomplished by dividing the spectrum into 0.02-ppm (or larger when indicated) regions over which the signal was integrated to obtain the intensity. The PCA was performed using Simca-P 12.0 (Umetrics, Umeå, Sweden).

## Results and Discussion

### Interactions of apo BLA with surfactants

Apo (Ca^2+^-free) BLA was titrated with the anionic detergent sodium dodecyl sulfate (SDS), the zwitterionic di-hexanoyl-phosphatidyl choline (C_7_PC), and the neutral decyl maltoside (DM). The titrations were followed by 1D ^1^H NMR. The 12 to 6.5 ppm region was used to avoid solvent signals. It was divided into 0.02 ppm bins, normalized by its total intensity, and analyzed by PCA.

**Figure 1:**
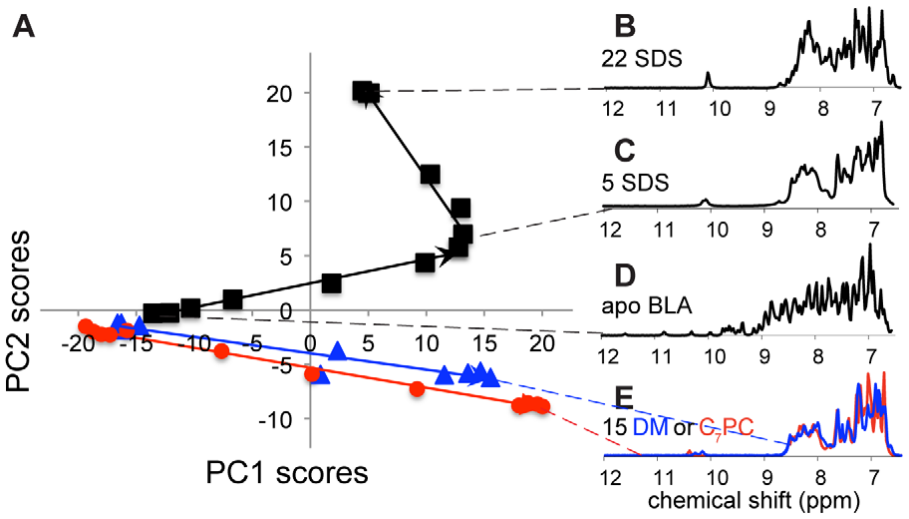
GPS NMR overview of the titrations of apo BLA with SDS, C_7_PC, and DM. GPS NMR 2D score plot (A) for titrations of apo BLA with SDS (black), C_7_PC (red), and DM (blue). PC1 and PC2 account for 56 and 19% of the variance between spectra, respectively. (B–E) NMR spectra of selected states: (B) SDS-induced state obtained above 22 detergents/protein, (C) SDS-induced state at 5 detergents/protein, (D) detergent-free folded state, and (E) C_7_PC and DM-induced states obtained at detergent/protein ratios >15.

The two first PCs in the GPS NMR 2D score plot (Fig. 1A) summarize 75% of the spectral changes for the three titrations. The SDS titration (Fig. 1A, black) can be described by two straight lines with a turning point around 5 SDS/BLA and the final state at 22 SDS/BLA. The straight lines suggest that the corners represent well-defined states. This is in good agreement with dialysis [11] and calorimetric [9] measurements The score plot can be interpreted as a phase/folding diagram where motion along the first titration line represents transition from the native state to the first SDS-induced state and motion along the second line represents transition from the first to the second SDS-induced state. The distribution of protein states at a given point on the lines is characterized by the distance to the two ends.

**Figure 2:**
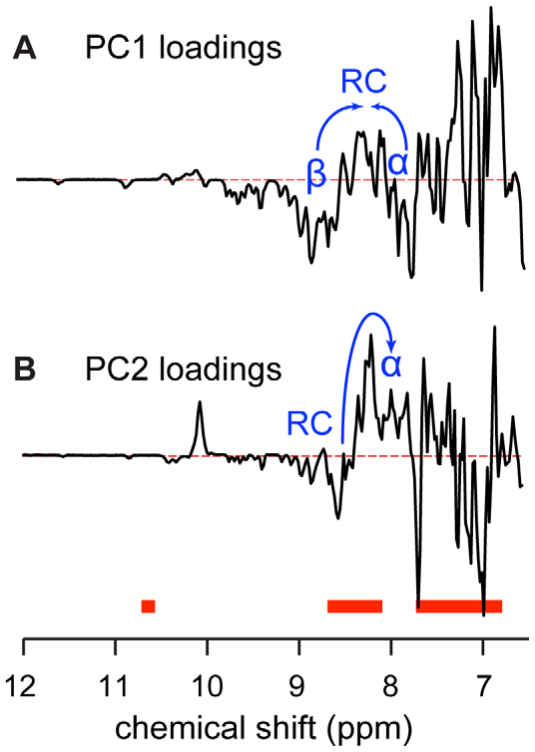
GPS NMR loading plots for the titrations of apo BLA with SDS, C_7_PC, and DM. Loadings are shown for PC1 (A) and PC2 (B). The thick red lines show typical random coil chemical shifts for from left to right: tryptophan sidechain amide, backbone amides, and aromatic sidechains [12]. Blue arrows indicate changes in the backbone amide region.

**Figure 3:**
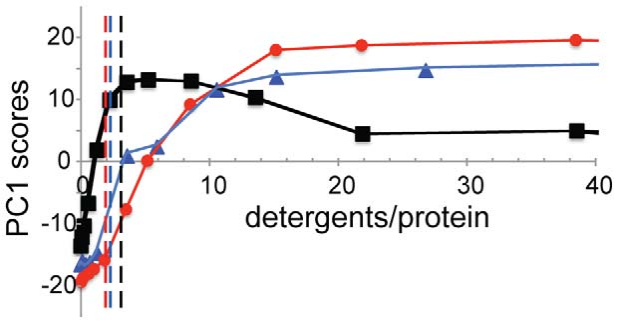
GPS NMR scores as a function of added SDS, C_7_PC, or DM. PC1 scores for SDS (black), C_7_PC (red), and DM (blue) versus detergent concentration. The dashed lines indicate the CMCs of the surfactants in absence of protein [9].

The spectral changes along PC1 and PC2 are described by the loadings in Fig. 2. The PC1 loadings (Fig. 2A) show that with increasing PC1 scores (first SDS association step), the dispersion of the amide and aromatic regions is lost as marked by decreasing signals outside and increasing signals inside the random coil regions [12]. Though effects such as hydrogen-bond formation also are important for the amide proton chemical shifts, lower shifts are indicative of α-helix and higher shifts of β-strand [13]. The changes in the dispersion of the amide proton signals (blue arrows in Fig. 2A) thus indicate loss of both a-helical and b-sheet secondary structure. There is also a relative signal gain in the aromatic side chains relative to the amides. Altogether this reveals a loss of tertiary structure and conformational exchange on the µs-ms timescale for the amides. The PC2 loadings (Fig. 2B) show that with increasing PC2 scores (second association step), the amide signals move towards lower chemical shifts (blue arrow). The tryptophan amides form one strong signal, indicating a significant shift in the timescale of chemical exchange. The observation of these two processes agrees with the unfolding of the tertiary structure and subsequent α-helix formation seen by fluorescence and CD spectroscopy [9], [11], [14]. Complementary information about the structural changes that occur can be obtained by inspection of the spectra closest to the “pure” states in the three corners of the graph (Fig. 3).

The turning point at 5 SDS/BLA (Fig. 1 and 3) agrees well with the finding that 4 SDS molecules remained bound after several days of dialysis [11], and the calorimetric observation that 4.4 SDS molecules bind to BLA in the first unfolding transition [9]. Calorimetric measurements also identified a total of 25 SDS molecules bound at the end of a second transition[9], which agrees with the 22 SDS molecules required for the scores to reach a plateau (Fig. 3).

Apo BLA shows a well-dispersed spectrum with narrow signals (Fig. 1D) corresponding to a reasonably well-folded protein. Apart from the lost dispersion, the first SDS-induced state has significantly broadened signals, especially in the amide region (Fig. 1C) indicative conformational exchange on the µs-to-ms timescale. The second SDS-induced state is indeed better dispersed and has linewidths that are equal to or lower than in the absence of SDS (Fig. 1B) in accordance with a regain of structure and/or a significant shift in the timescale of chemical exchange.

An additional SDS-titration with deuterated EDTA and SDS was obtained in order to study effects on the aliphatic regions of the spectra that are otherwise covered by solvent protons (not shown). The chemical shift dispersion in the aliphatic region is drastically decreased in the first transition, which confirms the loss of tertiary structure. The second transition only causes minor rearrangements for the aliphatic region, confirming that no tertiary interactions are present in the second SDS-induced state. None of the SDS-induced states show upfield-shifted aliphatic signals, indicating that very few tertiary interactions occur in any of these states (not shown).

The scores of the titrations with C_7_PC (Fig. 1A, red) and DM (Fig. 1A, blue) are well described by one line each with directions similar to the first step in the SDS titration. The neutral and zwitterionic surfactants do not seem to transform the protein into a third state, and the spectra for the final states are very similar for the two surfactants (Fig. 1E), in agreement with previous studies [9].

Titration curves along PC1 for SDS, C_7_PC, and DM as a function of the number of surfactant molecules per protein are shown in Fig. 3. Note the biphasic appearance of the titration curve of SDS. While the anionic SDS induces changes below CMC, the neutral DM and zwitterionic C_7_PC only induce detectable changes above CMC, in agreement with previous observations [9].

### Multiple perturbations

**Figure 4:**
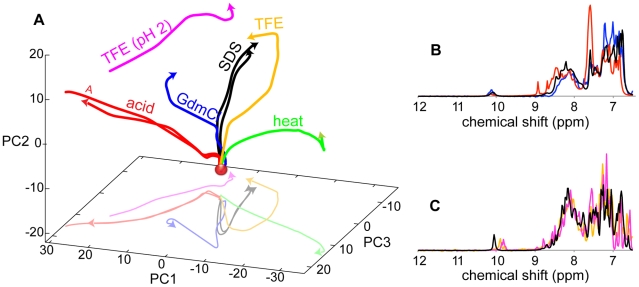
GPS NMR overview of apo BLA perturbed by addition of SDS, GdmCl, or TFE, by changes in pH, or heating. GPS NMR 3D score plot (A) for titrations of apo BLA with SDS (0–56 mM; black), pH (pH 7.0–2.0; red), GdmCl (0–3 M; blue), TFE (0–50%; orange), TFE at pH 2.0 (0–50%; pink), and heating (283–333 K; green). SDS titrations at 700 and 400 are included. The lighter lines show projections on the PC1-PC3 plane. The scores of pure apo BLA and the A-state are indicated by a red dot and an *italic A*, respectively. Colored numbers indicate turning/end-points of the various titrations. (B, C) Overlays of NMR spectra of partially folded states (B) induced by 5 SDS molecules/protein (black), 1.6 M GdmCl (blue), and addition of acid to pH 2 (red), and (C) by 22 SDS/BLA (black), 50% TFE (orange), and 50% TFE at pH 2 (pink).

To explore the wealth of folds sampled by BLA and to position the SDS-induced states in a more general conformational landscape, we expand our GPS NMR mapping with other types of perturbations. These include additions of acid and base, the denaturant guanidinium chloride (GdmCl), the organic solvent trifluorethanol (TFE), and heating (thermal denaturation) to 60°C. These perturbations were analyzed together with the SDS-induced ones in a single PCA. The GPS NMR 3D score plot in Fig. 4A accounts for 67% of the variations between all spectra. GPS NMR reveals numerous partially folded BLA states, the formation of which can be tracked in Fig. 4A. Addition of acid (Fig. 4A, red) induces the classical molten globule A-state at pH 2.6 [15]. The kink in the curve around pH 4.5, close to the isoelectric point, indicates the presence of another state at this pH. Addition of GdmCl (Fig. 4A, blue) produces a partially folded state at 1.6 M and a set of unfolded states starting at 2.2 M [16]. Heating (Fig. 4A, green) to 60°C gradually moves the protein away from the folded state but in a different direction.

GPS NMR allows similarities between structural transitions to be assessed. Addition of 1.6 M GdmCl moves the spectra along a path similar to the first step in the SDS titration (Fig. 4A). The spectra at 5 SDS/BLA and 1.6 M GdmCl are compared in Fig. 4B. Addition of TFE brings the protein along a path that, despite divergences in the second half of the titrations, is very similar to that induced by SDS. The two states end up quite close to each other. The similar effects of SDS and TFE are noteworthy considering their similar effects in various protein systems, where they both induce β-strand formation at lower concentrations and α-helix formation at higher [17], [18], [19], [20], [21].

Similarly structured parts in the A-state and the GdmCl-induced partially folded state are indicated by similar hydrogen exchange patterns [22]. Here, however, addition of acid and Gdm-Cl take the protein along different paths (Fig. 4). The different paths are reflected by the three major spectral differences between the A-state and those of the SDS- and GdmCl-induced states (Fig. 4B): two sharp signals below 9 ppm indicate that there are regions in the A-state that are governed by a different structural/dynamic regime than in the other two states. An intense signal at 7.6 ppm, and a slight down-field shift of the spectral envelope can be explained by changes in protonation and pH effects.

Addition of TFE at pH 2 brings the protein from the A-state to a position not far from the final state induced by TFE and SDS at neutral pH (Fig. 4A), in agreement with observed similarities in TOCSY spectra [14]. The similarities of final states induced by addition of TFE to BLA in neutral and acidic solutions suggest that the effect of pH is limited in the presence of 50% TFE. The acid-unfolded A-state and the TFE-induced state have been found to be similar in many ways [15], [23].

### Sensitivity, resolution, and robustness of the analysis

**Figure 5:**
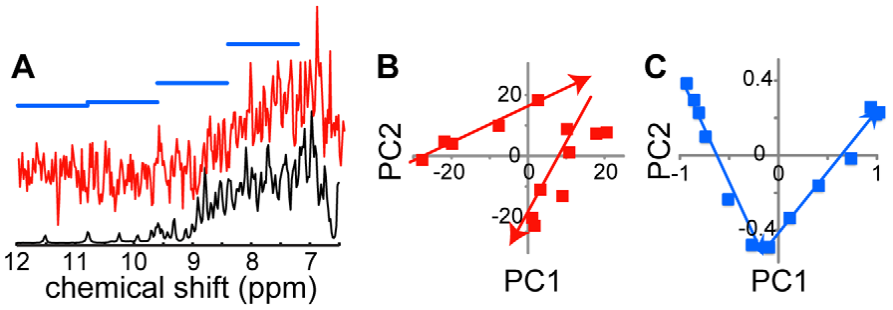
GPS NMR score plots from spectra with very low signal-to-noise or very low resolution. (A) Spectra of apo BLA: 1.2 mM acquired with 128 scans described using 275 data points (0.02 ppm; thin black line), 1.2 µM acquired with 4 scans described using 275 data points (0.02 ppm; thin red line), or 1.2 mM acquired with 128 scans described using 4 data points (1.2 ppm bins; thick blue lines), and GPS NMR 2D score plots for the titration of BLA with SDS (B) using the data with decreased concentration and acquisition time and (C) using the data with decreased resolution. PC1 and PC2 explain 31 and 22%, and 86 and 14% of the variance between spectra, respectively.

Data were with few exceptions phased and baseline corrected automatically. The data used above were acquired using 1.2 mM BLA with 128 scans in 5 min. To test the sensitivity of the analysis we performed PCA on the data from the SDS titration with artificially decreased signal-to-noise ratio. Both reduction in the sensitivity by up to a factor of 200 and acquisition of data with 4 scans in 15 s on 12 µM BLA at 800 MHz using a cryogenic probe (Fig. 5) results in at least two significant PCs and a well-defined turning point. To explore the need for spectral resolution, we performed PCA on the SDS data using larger bins. Bin widths up to 0.1 ppm resulted in virtually identical results, and bin widths up to 1.2 ppm (4 data points) resulted in at least two significant PCs and the turning point around 5 SDS/BLA (Fig. 5). Negligible effects of magnetic field strength are indicated by virtually identical scores for SDS titrations performed at 400 and 700 MHz (Fig. 4A). Sensitivity and resolution are critical for any experimental method, but the analysis in Fig. 5 clearly demonstrates that GPS mapping of BLA may be established at radically more forgiving conditions for spectral sensitivity and resolution than typical for NMR applications. The red spectrum in Fig. 5A represents data recorded at a concentration of 12 µM BLA using 1/32 of the experimental time (4 scans for each of the 13 spectra) used for the black higher concentration spectrum (1.2 mM) used in Fig. 1. Data analysis of spectra with this remarkably low sensitivity provides a GPS NMR score plot as illustrated in Fig. 5B, clearly reflecting the same three-stage (two-line) folding process of BLA upon titration with SDS. The blue horizontal lines (Fig. 5A), representing the 1.2 mM data integrated into 4 bins of width 1.2 ppm also leads to a three-stage (two-line) GPS NMR folding map. In combination these two plots reveals that GPS NMR works at concentrations far below what would be required for analysis involving spectral assignment and protein NMR in general, and demonstrates that even low-resolution spectra (or spectra displaying a high degree of spectral overlap) offers the potential for GPS mapping, such as very large or dilute systems, systems undergoing chemical exchange, and spectra acquired using solid-state or low-field NMR [24].

An additional strength of GPS NMR is that isotope labels are not needed, so that protein from (or in) the natural source can be used. This is a great advantage for systems for which recombinant expression is laborious, costly, or leads to proteins with altered properties compared to the authentic protein, which is the case for BLA [14], [25].

**Figure 6:**
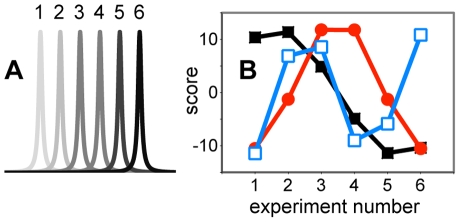
The impact of moving signals on the PCA scores. (A) A signal moving in 6 equidistant steps from left (light gray) to right (black). (B) The PCA scores for the first 3 components (PC1, black squares; PC2, red circles; PC3, open blue squares) as a function of spectrum number. Notice the similarity to standing waves.

GPS NMR is sensitive to the distribution of intensity along the chemical shift axis, which depends on the distribution of structural characteristics and the relative dynamic features. Unless prior data treatment is used to extract a moving signal in terms of its position alone (cf. Ref. [6]), PCA does not allow us to follow signals as they move, *i.e.* change chemical shift. In fact, the presence of a strong moving signal results in a large number of PCs with scores that vary with the experiment number like standing waves of increasing order (Fig. 6). It is therefore very important to examine the scores and loadings for each PC before interpreting the individual PC as a structural change. In practical terms, two options exist in the interpretation of PC from spectra dominated by moving signals: (*i*) Make the bin width larger (cf. Fig. 5) so that most movements will occur inside the bins, or (*ii*) discard regions of the spectra where the movements occur.

The normalization to total intensity is important to eliminate trivial separations due variations in amount of sample or other experimental parameters. Different normalization schemes including separate treatment of different regions, and/or including other spectral regions were tested. In all cases the general trends in this analysis agree.

### Comparison to other methods

Time-resolved folding and unfolding of α-lactalbumin [22], [26], [27], [28], [29] and other proteins [30] have been studied by conventional 1D and 2D NMR. Data from these studies were evaluated by integrating individual peaks and fitting time constants locally or globally. When using PCA, the PCs are linear combinations of the different processes and the time constants can be calculated from the time-dependence of the scores of the different PCs. As discussed above, there is a significant increase in S/N, which allows shortening of the experimental time and/or a decrease in the amount of sample used. This may be an important improvement when studying more complex reactions. The data analysis in GPS NMR is also fast. Analysis of a new system takes a few hours irrespective of the number of spectra used.

### Future directions

The method can be further enhanced by inclusion of *T*_1_, *T*_2_, or diffusion edited experiments to put more weight on dynamic aspects, or STD [31], exchange-transferred NOE [32], or similar experiments to probe for interactions. The use of a cryogenic flow or microprobe system in combination with automatic mixing of a large number of samples would allow a full map of the folding space of proteins. The results of this could give important information about the states spanned in the (soluble part of the) promiscuous life of proteins in their natural environment as well as screening a molecule of study for optimal solution conditions.

### Conclusions

GPS NMR mapping allows the systematic study of the states sampled by a protein exposed to different perturbations by the application of simple 1D NMR spectra in combination with multivariate analysis. The aim has not been to exploit all spectral information that may be obtained by NMR spectroscopy to establish atomic-resolution structural information, as has been demonstrated for decades, but rather to demonstrate the capability of NMR to be used as a high-throughput method which from simple 1D spectra and fast data analysis may provide global information about protein folding. Our study of α-lactalbumin clearly demonstrates that the method fast and reliably can identify a number of known thermodynamically stable partially folded BLA states and the transitions between them. We have shown that by analysing many spectra simultaneously, the method works at very low signal-to-noise levels relative to traditional analysis of NMR spectra, and can treat data with very limited spectral quality. This opens up the possibility to study proteins at low concentrations and in complex mixtures such as their native environments, for analysis of data of different origins such as different magnetic fields and potentially for the use of less expensive low-field NMR instrumentation. We envisage that GPS NMR will become a powerful tool in protein characterization, the possibilities of which we have just started to explore.
